# Additive manufacturing of silica glass using laser stereolithography with a top-down approach and fast debinding

**DOI:** 10.1039/c8ra02428f

**Published:** 2018-05-03

**Authors:** Chang Liu, Bin Qian, Xiaofeng Liu, Limin Tong, Jianrong Qiu

**Affiliations:** School of Materials Science and Engineering, Zhejiang University Hangzhou Zhejiang China; State Key Laboratory of Modern Optical Instrumentation, School of Optical Science and Engineering, Zhejiang University Hangzhou Zhejiang China qjr@zju.edu.cn

## Abstract

Additive manufacturing of silica glass by stereolithography is a new technology. Improving the efficiency of this technology is an important issue. In this work, a stereolithography system using top-down approach and a compatible suspension for this approach was developed. The debinding behavior of the green part (the solidified suspension) was discussed in detail based on the results of thermogravimetric analysis and Fourier transform infrared absorption spectroscopy. A fast heat treatment within 16 hours was adopted for debinding and sintering of the green part which took only 1/3 of the time of previous report. Designed glassware which could not be processed by conventional technologies was manufactured in this way, and the products showed similar properties with fused silica, as confirmed by X-ray diffraction and UV-vis-IR spectrometer.

## Abstract 2

A new approach for glass additive manufacturing with stereolithography that raises the efficiency in a great extent.

## Graphical abstract

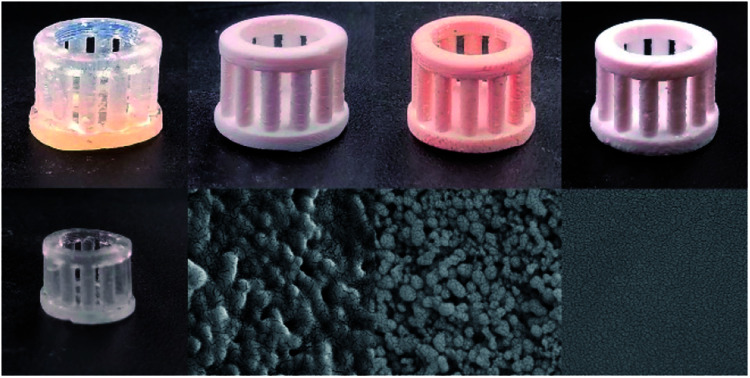

## Introduction

Glass is an important material for its hardness, optical transparency, chemical stability and heat resistance, with diverse chemical, medical, optical and photonics applications.^1–4^ However, due to its hard and brittle character, it is impossible to attain complex glassware with conventional manufacturing technologies. This strongly limits the accessibility of glass for modern applications, such as micro-optic and microfluidic devices. Additive manufacturing (AM), also designated as 3D-printing, has changed the way for conventional industrial manufacturing.^5^ Since AM was first documented by Charles Hull in 1984,^6^ this technology has been applied in the manufacturing with various materials, including hard and brittle materials such as titanium alloys and silicon carbide ceramics.^7,8^ Thus, AM is considered as a potential technology for modern glass manufacturing.

In recent years, several AM technologies were developed for glass, including selective laser melting, filament-fed, fused deposition modeling and binder jetting.^6,9–11^ However, glassware manufactured with these technologies suffered from opacity and low resolution. Stereolithography (SL), a technology which uses photopolymerization of powders filled photosensitive suspensions with the following debinding and sintering, has already gained success in ceramics AM.^12^ The successful fabrication of transparent glass *via* sintering makes it possible to use a similar SL technology in glass AM.^13,14^

The first SL technology for glass AM was reported by Kotz *et al.*, in which transparent glassware with high resolution was manufactured.^15^ However, the bottom-up approach they adopted suffers from separation force and gravitational force, which seriously raises the difficulty in this AM technology.^16^ As a result, the bottom-up approach is seldom used in the SL systems for large scale industrial producing. To improve the efficiency, the top-down approach is preferred for its capability in large scale manufacturing, though it has a strict requirement for the fluidity of the suspensions.^17^ Otherwise, heat treatment takes about 50 hours, which was 20 times longer than the photopolymerization, making it a strong limitation to the manufacturing efficiency. Thus, shortening the heat treatment is also necessary to improve glass AM with SL.

In this work, a UV curable suspension with low viscosity which was suitable for the top-down approach was prepared, and a top-down SL system with a 355 nm laser source and a scan-mirror system was developed. Computer aided designed green parts (the solidified green part) were manufactured with this system. A heat treatment which was completed in 16 hours was adopted according to the thermogravimetric analysis (TGA) to transform the green parts to transparent glassware. The debinding behavior during the heat treatment process was discussed in detail based on the results of Fourier transform infrared absorption spectroscopy (FTIR) and scanning electron microscopy (SEM). As confirmed by X-ray diffraction (XRD) spectra and UV-vis-infrared transmittance spectra, the properties of the sintered glass were comparable with that of commercial fused silica.

## Experimental

### Materials

2-Hydroxyethyl methacrylate (HEMA, 99%), poly(ethylene glycol) diacrylate 200 (PEGDA 200), diethyl phthalate (99.5%), 4-methoxyphenol (MEHQ, 99%), 2,2-dimethoxy-2-phenylacetophenone (DMPA, 99%) and isopropyl alcohol (IPA, 99.5%) was provided by Aladdin, China. Amorphous silica nanoparticles (Aerosil OX50) were provided by Evonik, Germany. Tinuvin 1130 was provided by Basf, Germany.

### Preparation of the suspension

**Fig. 1:**
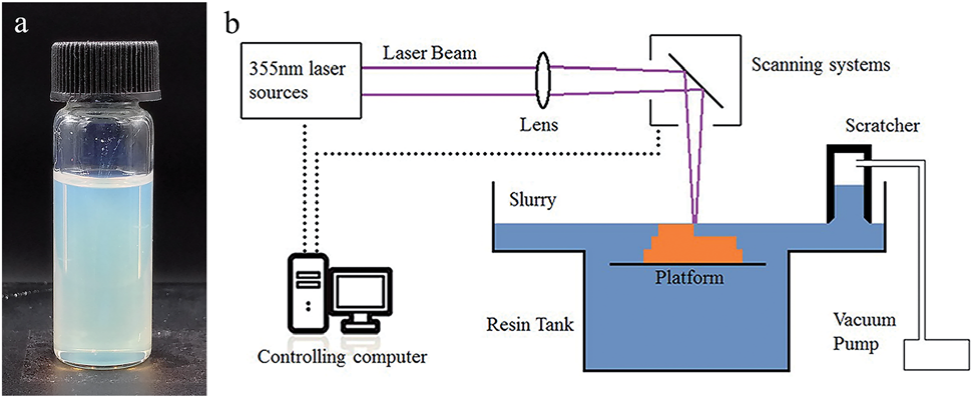
(a) Photograph of the UV curable suspension. (b) Schematic illustration of the SL system for glass AM.

29.5 wt% HEMA (reactive monomer), 3.7 wt% PEGDA 200 (crosslink agent) and 13.1 wt% diethyl phthalate (plasticizer) were mixed to form a homogeneous solution. 53.7 wt% silica nanoparticles were added to the solution in 50 doses, and were dispersed with a dissolver (D500, Dragonlab) after each dosing. Afterwards, 0.4 wt% DMPA (photo initiator), 0.4 wt% MEHQ (inhibitor) and 0.3 wt% Tinuvin 1130 (photo absorber), which was relative to the monomers, were dispersed in the mixture, and a homogeneous suspension (Fig. 1a) was attained.

### Manufacturing process

A 355 nm laser (pulse355-0.5, Inngu Laser, China) with a beam waist of about 120 μm on the focal plane and a scan system (GO5-YAG-10-D, JCZ Technology) composed of two scan mirrors were used to cure a certain pattern on the suspension surface. A substrate was set in the slurry tank, which was driven by a motor to move along the vertical direction. A scratcher with a negative pressure chamber was used to recoat and flatten the liquid surface after each layer was cured (Fig. 1b). The green parts were immersed into isopropyl alcohol for 1 minute to remove the surface attachment.

### Heat treatment

A tube furnace (GSL-1400X, Kejing Materials Technology, China) was used in the heat treatment. The green parts were loaded into a corundum crucible and they were heated up to 500 °C in air for debinding. Afterwards, a vacuum pump was used to remove the gas, and the furnace was heated up to 1250 °C for sintering.

### Characterization

Rheology of the suspension was measured with a rotational rheometer (RS6000, HAAKE, America) at 30 °C. XRD spectra were measured with a multi-crystal XRD spectrometer (D/MAX 2550/PC, Rigaku, Japan). TGA and differential thermal analysis (DTA) were done by a differential scanning calorimeter with a thermal balance (SDT Q600, TA Instrument, USA). The transmittance spectra were measured with a UV-vis-infrared spectrometer (U-4100, Hitachi, Japan). The microscopic pictures were shot with a scanning electron microscope (Utral 55, Carl Zeiss Jena, Germany), and the samples were coated with a layer of gold *via* sputtering. The FTIR spectra were measured with a Fourier transform infrared spectrometer (TENSOR27, Bruker, Germany).

## Results and discussion

**Fig. 2:**
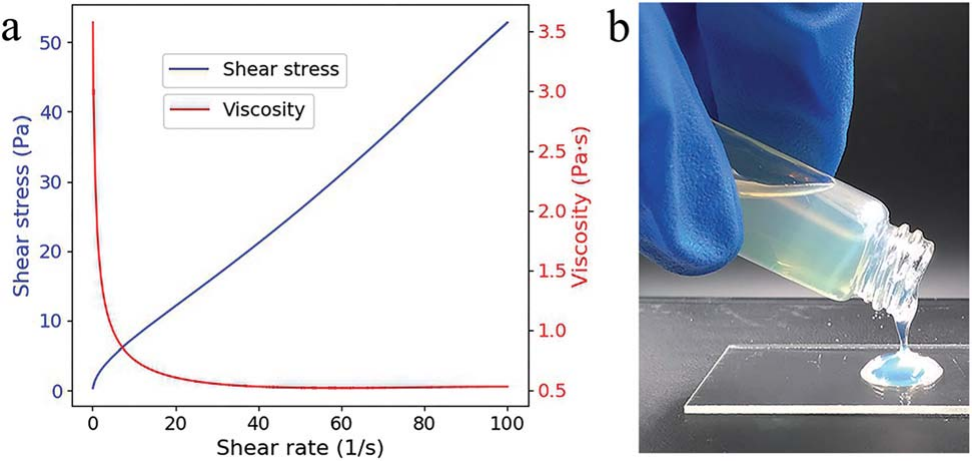
(a) Viscosity (red line) and shear stress (blue line) as a function of the shear rate. (b) The fluidic behavior of the composite.

The viscosity and the shear stress depend on the shear rate and shows a typical shear thinning phenomenon (Fig. 2a), which is probably resulted from the decreased relative contribution of entropic forces.^18^ Even at low shear rate (0.103 s^−1^), the viscosity (3.572 Pa s) is acceptable for top-down SL as the fluidity is quite good (Fig. 2b). No additive dispersant is needed in this suspension because the reactive monomer HEMA has high solubility for SiO_2_ powders.^14^

**Fig. 3:**
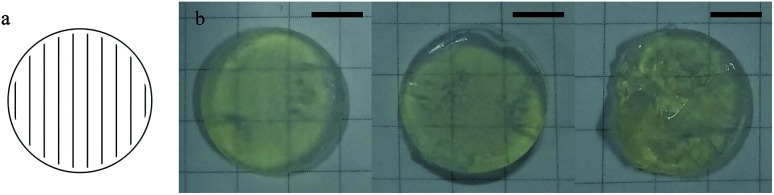
(a) Illustration of the scan path for a cycloidal pattern (the red line represents the hatch distance), (b) photos of the green parts (cylinder with diameters of 14 mm and thicknesses of 1.4 mm) fabricated under different SL parameters (from left to right, 60 mW 46 mm s^−1^, 130 mW 100 mm s^−1^ and 200 mW 154 mm s^−1^, scale bar, 2.5 mm).

For the laser SL process, several parameters including layer thickness, hatch distance, laser power, scanning speed were decided *via* experiments. The layer thickness was set to 100 μm, which was decided considering the balance between manufacturing efficiency and precision. To cure a certain pattern for each layer, the laser spot was moved along parallel lines that fill the pattern (Fig. 3a). The distance between the adjacent lines, which is defined as hatch distance, was set to 70 μm according to the laser beam waist.

The laser power and the scanning speed are tunable parameters which together decide the exposure on the unit surface of the suspension by:1
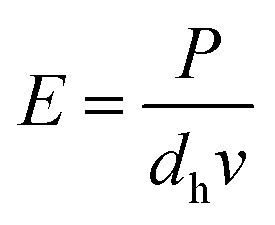
where *P* is the laser power, *d*_h_ is the hatch distance, and *v* is the scanning speed. The curing depth depends on the suspension properties and the exposure according to the following formula:^19^

2 *C*_d_ = *D*_p_ ln(*E*/*E*_c_)

where *C*_d_ is the curing depth, *E* is the exposure on the composite surface, *E*_c_ is the critical exposure of the composite, *D*_p_ is the depth of penetration. As *D*_p_ is decided by the suspension properties, which was fixed, the exposure *E* becomes the main factor contributing to the curing depth, which should be a little deeper than the layer thickness. Based on sufficient experimental trials, the suitable exposure for the SL is 1.86 J cm^−2^.

Not all the combinations of laser power and scanning speed that satisfies *E* = 1.86 J cm^−2^ are suitable for the SL technology. According to the experiments, relative low laser power and low scanning speed are favorable to reach a complete green part rather than to be destroyed by the scratcher (Fig. 3b). Although higher laser power and higher scanning speed may result in higher degree of polymerization,^20^ the higher radical concentration and shorter reaction period may cause low polymer chain length which reduce the strength of the green parts.^21^ However, lowering the scanning speed will lower the efficiency of manufacturing, and considering the balance of the manufacturing quality and efficiency, 60 mW and 46 mm s^−1^ for laser power and scanning speed were adopted.

**Fig. 4:**
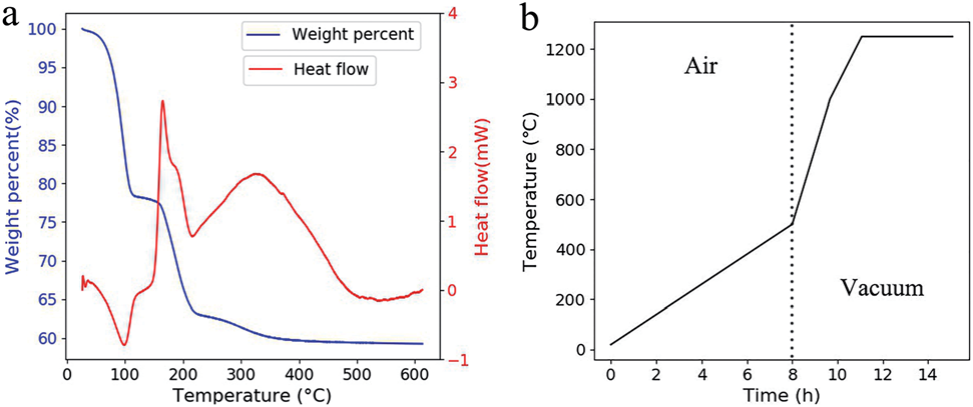
(a) TGA and DTA curves of the green part. The temperature was raised at 2 °C min^−1^. (b) The adopted temperature profile for heat treatment.

Three weight loss steps were observed at 50–120 °C, 160–230 °C and 260–350 °C in TGA (Fig. 4a). Compare with the reported work,^15^ these weight-loss steps appeared at much lower temperature. At the first weight-loss step, a valley appeared in DTA which meant an evaporation process happened. Compares with oxidation, evaporation process is much safer as the temperature is much controllable in this endothermic process. This evaporation led to the formation of penetrating tunnels which will improve material transport out of the green parts.^22^ Otherwise, the porous structure has more space for the expansion of the polymer during the following heat treatments. This makes it possible to shorten the debinding process as higher heat-up rates can be adopted and no holding time is needed. The time for the adopted heat treatment is less than 16 hours in total (Fig. 4b).

**Fig. 5:**
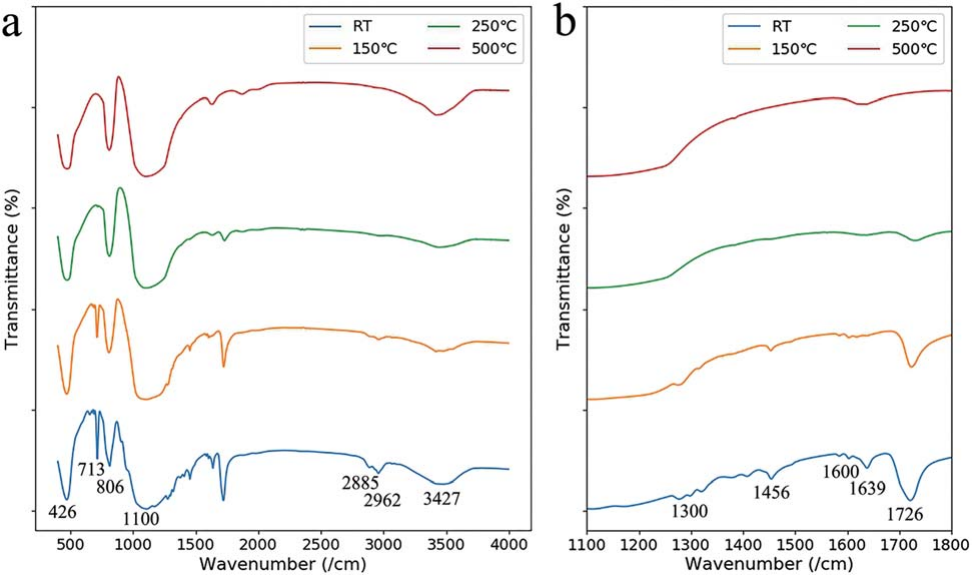
(a) IR absorption spectra of the green parts after heat treatments at different temperatures (room temperature (RT), 150 °C, 250 °C, and 500 °C). (b) The IR absorption spectra in the range of 1100–1800 cm^−1^.

The debinding behavior at each weight loss step was discussed in detail by comparing the IR absorption spectra of the green part with heat treatments at different temperatures (Fig. 5). At the first weight loss step from 50 °C to 120 °C, the absorption peak at 1639 cm^−1^ (C=C groups of HEMA and PEGDA) disappeared, and the peaks at 713 cm^−1^ ((CH_2_)_*n*_ groups) and 1726 cm^−1^ (C=O groups) receded.^23^ We infer that the weight-loss is mainly due to the evaporation of the unpolymerized monomers in this step, which is a result of the relative low polymerization rate due to short reaction period in laser processing.

At the second weight loss step from 160 °C to 230 °C, the IR absorption peaks around 1600 cm^−1^ (phenyl group of the diethyl phthalate), the peak at 713 cm^−1^, the peaks at 1456 cm^−1^, 2885 cm^−1^ and 2962 cm^−1^ (CH_3_ groups) and the peak at 1300 cm^−1^ (C–H bonds) disappeared.^23^ The disappearance of these peaks showed that the organic components were removed by oxidation at this temperature. However, the peak at 1726 cm^−1^ remained which is from carbonyl groups formed by incompletely oxidation.

As the temperature was raised to 500 °C, the oxidation of the carbonyl groups were finished as the IR absorption peat at 1726 cm^−1^ disappeared. The absorption at 426 cm^−1^, 806 cm^−1^, 1100 cm^−1^ and 3427 cm^−1^ were from Si–O–Si, Si–O and Si–OH groups. The absorption at 1634 cm^−1^ was from the O–H bond in H_2_O molecules which was adhesive to the porous SiO_2_ structure.^24,25^ The remaining SiO_2_ of about 59 wt%, more than that in the liquid suspension, was due to the dissolution of the unpolymerized monomers into IPA when removing the attachment.

**Fig. 6:**
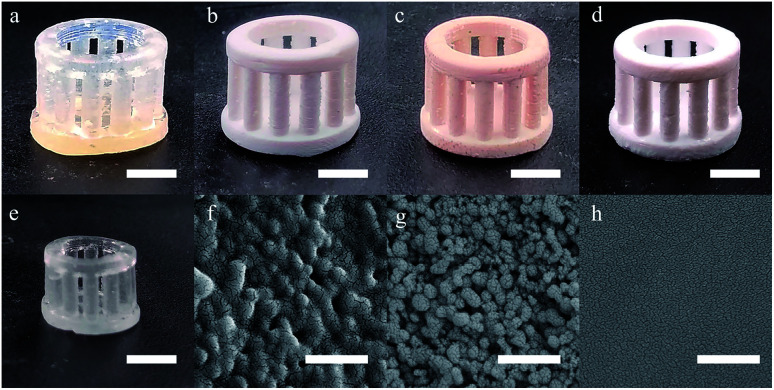
(a–e) Photographs of the samples after heat treatments at room temperature, 150 °C, 250 °C, 500 °C and 1250 °C (scale bar, 5 mm). (f–h) SEM images of the samples after heat treatments at room temperature, 500 °C and 1250 °C (scale bar, 500 nm).

Before heat treatment, the green part made by SL was transparent as the indices matching of the SiO_2_ powders and the polymers (Fig. 6a). The SiO_2_ nanoparticles coated by polymers were observed with SEM (Fig. 6f). After the heat treatment at 150 °C (Fig. 6b), the green part became opaque which was resulted from the light scattering of the inside tunnels. At 250 °C, the carbonization occurred, leading to the brown coloration of the green parts (Fig. 6c). At 500 °C (Fig. 6d), the brown color faded as the carbonyl groups were removed by oxidation, and the aggregation of SiO_2_ nanoparticles was observed with SEM (Fig. 6g). After being sintered at 1250 °C, a transparent glassware with a linear shrinkage of 28.6% was observed which was near the theoretical value (Fig. 6e), however, no obvious deformation was observed and a flat surface without pores was observed with SEM (Fig. 6h).

**Fig. 7:**
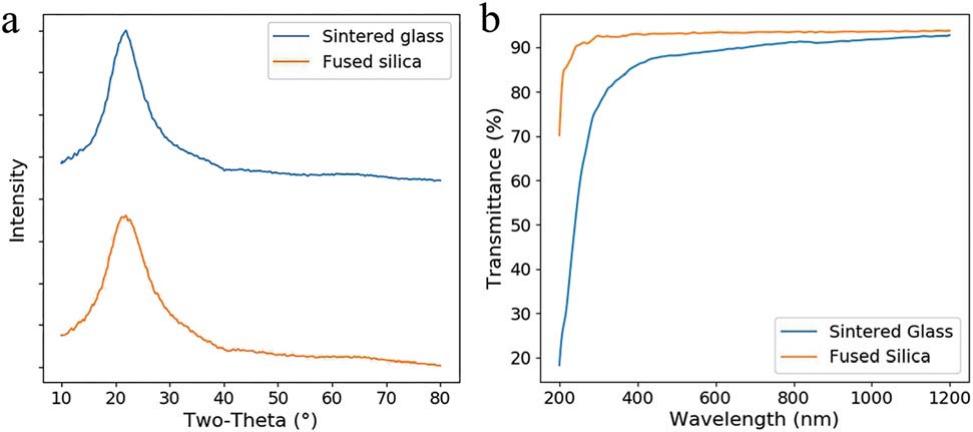
(a) XRD spectra of the sintered glass and the fused silica. (b) The transmittance spectra of the sintered silica glass and fused silica (both were thinned and polished, the thickness was 0.8 mm).

In the XRD spectra (Fig. 7a), no sharp diffraction peak of the sintered glass was observed, which is similar as fused silica. This result confirms that no crystallization occurs during the heat treatment. Lower transmittance and red-shifted absorption edge are observed by comparing the fused silica and the sintered glass in the transmittance spectra (Fig. 7b). This difference is probably caused by the scattering from the defects in the sintered glass. However, it is tolerable since high transmittance (>80%) was achieved in the region from 400 nm to 1200 nm.

## Conclusions

A top-down approach SL for glass AM with a 355 nm laser was developed, and a SiO_2_ nanoparticles filled low viscosity suspension was prepared. Manufacturing parameters were optimized for SL and glassware with designed structures was manufactured. Because of the low polymerization rate, which was due to the short reaction period in laser processing, an evaporation of unpolymerized monomers started below 100 °C was observed. This evaporation built penetrating tunnels that improved material transport out of the green parts which gave the possibility to a fast heat treatment finished within less than 16 hours. The sintered glass showed similar properties as commercial fused silica. The products were free of crystallization and had more than 80% transmittance in the region of 400–1200 nm.

## Conflicts of interest

There are no conflicts to declare.

## Supplementary Material
